# Effect of diabetes duration and glycaemic control on 14-year cause-specific mortality in Mexican adults: a blood-based prospective cohort study

**DOI:** 10.1016/S2213-8587(18)30050-0

**Published:** 2018-06

**Authors:** William G Herrington, Jesus Alegre-Díaz, Rachel Wade, Louisa Gnatiuc, Raúl Ramirez-Reyes, Michael Hill, Martha Solano-Sánchez, Colin Baigent, Sarah Lewington, Rory Collins, Roberto Tapia-Conyer, Richard Peto, Pablo Kuri-Morales, Jonathan R Emberson

**Affiliations:** a Medical Research Council Population Heath Research Unit, Clinical Trial Service Unit and Epidemiological Studies Unit, Nuffield Department of Population Health, University of Oxford, Oxford, UK; b Clinical Trial Service Unit and Epidemiological Studies Unit, Nuffield Department of Population Health, University of Oxford, Oxford, UK; c School of Medicine, National Autonomous University of Mexico, Mexico City, Mexico

## Abstract

**Background:**

Diabetes is a cause of at least a third of all deaths in Mexican adults aged 35–74 years, with the excess mortality due mainly to vascular disease, renal disease, infection, and acute diabetic crises. We aimed to analyse the effect of diabetes duration and glycaemic control on death rate ratios (RRs) for these causes and to assess the relevance to cause-specific mortality of undiagnosed diabetes.

**Methods:**

About 100 000 women and 50 000 men aged 35 years or older from Mexico City were recruited into a blood-based prospective study between April 14, 1998, and Sept 28, 2004, and followed up until Jan 1, 2016, for cause-specific mortality. Participants who, at recruitment, reported any chronic disease other than diabetes and those who had missing data for HbA_1c_ or diabetes duration were excluded. We used Cox models to estimate the associations of undiagnosed or previously diagnosed diabetes (almost all type 2) with risk of mortality from vascular disease, renal disease, and infection, exploring among those with previously diagnosed diabetes the independent relevance of diabetes duration (<5 years, ≥5 to <10 years, or ≥10 years) and HbA_1c_ (<9%, ≥9% to <11%, or ≥11%). We also estimated the association of HbA_1c_ with mortality in participants without diabetes at recruitment.

**Findings:**

133 662 participants were aged 35–74 years and had complete data and no other chronic disease. 16 940 (13%) had previously diagnosed diabetes, 6541 (5%) had undiagnosed diabetes, and 110 181 (82%) had no diabetes. Among participants with previously diagnosed diabetes, glycaemic control was poor (median HbA_1c_ 8·9% [IQR 7·0–10·9]), and was worse in those with longer duration of disease at recruitment. Compared with participants without diabetes, the death RRs at ages 35–74 years for the combination of vascular, renal, or infectious causes were 3·0 (95% CI 2·7–3·4) in those with undiagnosed diabetes, 4·5 (4·0–5·0) for the 5042 participants with a diabetes duration of less than 5 years, 6·6 (6·1–7·1) for the 7713 participants with a duration of 5 years to less than 10 years, and 11·7 (10·7–12·7) for the 4185 participants with a duration of at least 10 years. Similarly, the death RRs were 5·2 (4·8–5·7) for those with HbA_1c_ less than 9%, 6·8 (6·2–7·4) for those with HbA_1c_ of 9% to less than 11%, and 10·5 (9·7–11·5) for those with HbA_1c_ of at least 11%. Diabetes was not strongly associated with the combination of deaths from other causes apart from acute glycaemic crises. Among participants without diabetes, higher HbA_1c_ was not positively related to mortality.

**Interpretation:**

In Mexico, the rates of death from causes strongly associated with diabetes increased steeply with duration of diabetes and were higher still among people with poor glycaemic control. Delaying the onset of type 2 diabetes, as well as improving its treatment, is essential to reduce premature adult mortality in Mexico.

**Funding:**

Wellcome Trust, the Mexican Health Ministry, the Mexican National Council of Science and Technology, Cancer Research UK, British Heart Foundation, and the UK Medical Research Council Population Health Research Unit.

## Introduction

By 2045, an estimated 625 million people worldwide will be living with diabetes, compared with an estimate of 425 million for the year 2017.^1^ Most of the burden is and will continue to be borne by low-income and middle-income countries, where resources to treat diabetes and its complications might be less available than in high-income countries.^1^ However, evidence of the effect of diabetes on mortality has been derived largely from high-income countries, where patients might receive early diagnosis, have good access to medical care, and can achieve good glycaemic control. In such populations, diabetes almost doubles the all-cause mortality rate, with most deaths due to increases in vascular disease, infection, and some cancers,^2^, ^3^ and each 5 years' longer duration of diabetes is associated with about a 20% further increase in vascular risk.^4^, ^5^, ^6^

Mexico is an example of a large, upper-middle-income country in which obesity and diabetes are very common.^7^ Evidence from the Mexico City Prospective Study, which included 150 000 adults in Mexico City recruited between 1998 and 2004, showed that more than one in five had been diagnosed with diabetes by age 60 years, and glycaemic control in those diagnosed with diabetes was poor (a third had HbA_1c_ >10%).^8^ Furthermore, previously diagnosed diabetes was associated not with a doubling, but a quadrupling of the all-cause mortality rate during the subsequent 12 years, with the main excess mortality from renal and vascular disease, infection, and acute diabetic crises.^8^ In this report, our main objective was to analyse how diabetes duration and glycaemic control modify these death rate ratios (RRs) in the Mexico City Prospective Study, as well as to assess the relevance to cause-specific mortality of undiagnosed diabetes at recruitment. We also aimed to assess the relevance of HbA_1c_ to cause-specific mortality in participants without diabetes.

**Research in context**

**Evidence before this study**

Meta-analysis by the Emerging Risk Factors Collaboration showed that diabetes and increased blood glucose concentration (>6 mmol/L) are associated with premature mortality from non-vascular and vascular diseases. However, these data (from a total of 192 000 people, 23 000 of whom had diabetes) were collected from countries with generally good access to medical care and early diagnosis. It is unknown how diabetes duration and the more extreme blood glucose concentrations seen in countries with resource-limited health-care settings modify these associations.

**Added value of the study**

The Mexico City Prospective Study includes information and measurement of HbA_1c_ in almost 134 000 adults aged 35–74 years at recruitment, including about 23 000 with diabetes. During our 14 years of prospective follow-up, the deaths most strongly related to diabetes were those attributed to a vascular, renal, or infectious cause. Our results showed that for combined deaths from any of these three causes, the death rate ratio at ages 35–74 years for patients with diabetes compared with those without ranged from 3·0 for those with undiagnosed diabetes to 11·7 for those with diabetes of at least 10 years' duration. For a given time since diabetes diagnosis, poor glycaemic control (HbA_1c_ ≥9%) was associated with about twice the mortality risk as better control (HbA_1c_ <9%). The rate at which the rate ratios for vascular deaths increased with longer duration of diabetes (about a 20% further increase in the rate ratio per 5 years longer duration), was similar to what has been reported previously, but the rate of increase for renal death (about a 50% further increase in the rate ratio per 5 years longer duration) was somewhat larger.

**Implications of all the available evidence**

Strategies to both delay the onset of type 2 diabetes as well as improve glycaemic and associated risk factor control could substantially reduce the number of premature adult deaths in countries such as Mexico, where the resources to treat diabetes have not been able to keep pace with the growing obesity and diabetes epidemic.

## Methods

### Study design and participants

This study is a long-term follow-up analysis of the Mexico City Prospective Study; full details of the design and sampling methods of the study are reported elsewhere.^8^, ^9^ Briefly, between April 14, 1998, and Sept 28, 2004, households within urban areas of two districts of Mexico City (Coyoacán and Iztapalapa) were visited systematically and all residents aged 35 years and older were invited to join the study. Age, sex, socioeconomic status, lifestyle (eg, drinking, smoking, and physical activity), current medication, and medical history (including previously medically diagnosed diabetes and approximate year of diagnosis) were recorded. Height, weight, waist and hip circumferences, and seated blood pressure were measured and almost all (155 691 [97·5%] of 159 755) participants provided a non-fasting 10 mL blood sample. Participants who were aged 85 years or older and those who reported any other chronic disease at recruitment (vascular disease, chronic kidney disease, cancer, cirrhosis, or emphysema) were excluded from the main analyses, as were those with missing or implausible data for HbA_1c_, diabetes duration (if relevant), or any relevant confounder (listed in statistical analysis section), or with uncertain mortality linkage. Research ethics approval was obtained from the Mexican Ministry of Health, the Mexican National Council for Science and Technology, and the University of Oxford, UK. All the study participants provided written informed consent.

### Procedures

After collection, baseline blood samples were transported to the central laboratory in Mexico City, separated and frozen.^8^ Plasma and buffy coat samples were later shipped to Oxford, UK, for long-term storage. Buffy coat samples were analysed for HbA_1c_ in the Clinical Trial Service Unit's ISO17025-accredited Wolfson laboratories, using validated high-performance liquid chromatography methods^10^ on HA-8180 analysers with calibrators traceable to International Federation of Clinical Chemistry standards.^11^

Death registration in Mexico is reliable and complete, with almost all adult deaths certified medically and few attributed to unknown causes. For our analysis, deaths were tracked up to Jan 1, 2016, through electronic linkage to the death registry and probabilistic matching to study participants (with field validation in a subset confirming the reliability of the matching algorithm). The registry codes all diseases on the death certificate according to the International Classification of Diseases version 10.^12^ Study clinicians reviewed death certificates, accepting diabetes as the underlying cause only for deaths from acute diabetic crises.^8^

### Statistical analysis

We defined previously diagnosed diabetes as self-reported previous medical diagnosis at recruitment, use of any antidiabetes medication, or both, and defined undiagnosed diabetes as no previously diagnosed diabetes but an HbA_1c_ of 6·5% or higher (≥48 mmol/mol) at the single baseline assessment. Patients with diabetes onset at an age younger than 35 years who were taking insulin at recruitment were considered likely to have type 1 diabetes.

The main analyses examined the relevance of previously diagnosed and undiagnosed diabetes to death from vascular disease, renal disease (mainly chronic kidney disease, urosepsis, and acute kidney injury), and other infection (appendix), compared with individuals without diabetes. Analyses of the combination of all other causes of death apart from acute diabetic crises (neoplastic, cirrhotic, chronic obstructive pulmonary, and other, ill-defined, or external causes), and of all causes of death combined, were also done for comparison.

Most analyses were restricted to deaths at ages 35–74 years (ie, deaths that might be considered premature), but some analyses of deaths at ages 75–84 years were also done. Cox regression was used to estimate the separate relevance of previously diagnosed and undiagnosed diabetes at recruitment to mortality (relative to no diabetes) with attained age as the underlying time variable. For those without diabetes at recruitment, additional analyses examined the relevance of HbA_1c_ to mortality in three approximately equally sized groups. Cox models yield cause-specific log hazard ratios that provide a weighted average of the log death RRs in different time periods (which, irrespective of the proportional hazards assumption, provide useful summary statistics of the average death RRs during the study). Participants who did not die from the cause under study were censored at the earliest of death from an alternative cause, the end of the risk period under consideration (eg, age 75 years for analyses of risk during ages 35–74 years), or the end of follow-up for mortality (Jan 1, 2016).

In the main analyses, participants with previously diagnosed diabetes were subdivided by diabetes duration (<5, ≥5 to <10, or ≥10 years) or glycaemic control at recruitment (HbA_1c_ <9%, ≥9% to <11%, or ≥11%). In some analyses, those with diabetes duration less than 10 years or with HbA_1c_ of at least 9% were analysed as a single group without further subdivision. Analyses were adjusted for, or estimated separately by, age-at-risk (5-year categories), sex, location (two districts), education (university or college, high school, elementary school, or other), smoking (never, former, occasional, less than ten cigarettes per day, ten or more cigarettes per day), and anthropometry (height, weight, waist and hip circumferences, each split into four equally sized groups). Analyses by diabetes duration were standardised for glycaemic control and analyses by glycaemic control were standardised for diabetes duration (both to the average levels of those with previously diagnosed diabetes).

Assuming causality, estimates of the absolute excess risk of death from particular causes associated with previously diagnosed diabetes with poor glycaemic control (HbA_1c_ ≥9%), previously diagnosed diabetes with better glycaemic control (HbA_1c_ <9%), and undiagnosed diabetes were then estimated using a previously described method.^8^ We also calculated the proportion of each cause of death attributable to previously diagnosed or undiagnosed diabetes.

Sensitivity analyses included repeating the main analyses with additional adjustment for alcohol consumption, blood pressure, and physical activity, including data from participants who had a previously diagnosed chronic disease other than diabetes, and including those with missing covariate data through use of multiple imputation. Analyses were done with SAS version 9.3 and R version 3.0.1. All stated CIs are 95% CIs corresponding to a specified comparison between two groups. However, in figures showing death RRs by duration of diabetes or baseline HbA_1c_, group specific 95% CIs, which reflect the variance of the log risk only in that one group, are shown for every RR (including the reference group, which is assigned an RR of 1·0).^13^ A comparison of these two types of CI is in the appendix.

### Role of the funding source

The funders had no role in study design, data collection, data analysis, data interpretation, or writing of the report. The corresponding authors had full access to all the data in the study and had final responsibility for the decision to submit for publication.

## Results

Between April 14, 1998, and Sept 28, 2004, 112 333 households with at least one eligible resident were visited, with one or more participants agreeing to take part for 106 059 (94%) households. From these households, 159 755 participants were recruited. Of these, 8135 were excluded from our analyses because of previous chronic disease other than diabetes, and a further 10 448 were excluded because of missing data or because they were aged 85 years or older at the time of recruitment. Of the remaining 141 172 participants, 133 662 were aged 35–74 years at recruitment (table 1) and 7510 were aged 75–84 years (appendix).

**Table 1:** Baseline characteristics of 133 662 particpants aged 35–74 years at recruitment

		**No diabetes (n=110 181)**	**Undiagnosed diabetes (n=6541)**	**Previously diagnosed diabetes (n=16 940)**	**Previously diagnosed diabetes, by duration of diabetes**			**Previously diagnosed diabetes, by HbA**_1c_**, %**			**Overall (n=133 662)**
					<5 years (n=5042)	≥5 to <10 years (n=7713)	≥10 years (n=4185)	<9% (n=8475)	≥9 to <11% (n=4311)	≥11% (n=4154)	
**Age and sex**											
Age, years		49 (10)	54 (10)	57 (10)	54 (10)	57 (10)	62 (9)	59 (10)	57 (10)	55 (9)	50 (11)
Male		35 588 (32%)	2208 (34%)	5527 (33%)	1660 (33%)	2515 (33%)	1352 (32%)	2838 (33%)	1430 (33%)	1259 (30%)	43 323 (32%)
**Diabetes duration and severity**											
Duration of diabetes, years*		..	..	9 (7)	2 (2)	8 (3)	19 (6)	8 (7)	10 (8)	10 (7)	..
Onset age <35 years and insulin use at recruitment†		..	..	213 (1%)	11 (<1%)	54 (1%)	148 (4%)	50 (1%)	74 (2%)	89 (2%)	..
Mean HbA_1c_, %		5·5 (0·4)	8·6 (2·1)	9·1 (2·5)	8·5 (2·5)	9·3 (2·4)	9·4 (2·4)	7·0 (1·1)	10·0 (0·6)	12·4 (1·2)	6·1 (1·7)
Median (IQR) HbA_1c_, %		5·4 (5·2–5·7)	7·7 (6·8–10·3)	8·9 (7·0–10·9)	7·9 (6·4–10·3)	9·3 (7·3–11·1)	9·4 (7·5–11·1)	7·0 (6·2–7·9)	10·0 (9·5–10·5)	12·1 (11·5–13·0)	5·5 (5·3–5·9)
HbA_1c_ >9·0%‡		..	2367 (36%)	8465 (50%)	1970 (39%)	4184 (54%)	2311 (55%)	0 (0%)	4311 (100%)	4154 (100%)	10 832 (8%)
**Socioeconomic status and smoking**											
Resident of Coyoacán		44 735 (41%)	2111 (32%)	5719 (34%)	1768 (35%)	2167 (28%)	1784 (43%)	2934 (35%)	1440 (33%)	1345 (32%)	52 565 (39%)
Resident of Iztapalapa		65 446 (59%)	4430 (68%)	11 221 (66%)	3274 (65%)	5546 (72%)	2401 (57%)	5541 (65%)	2871 (67%)	2809 (68%)	81 097 (61%)
University or college educated		19 537 (18%)	627 (10%)	1312 (8%)	413 (8%)	664 (9%)	235 (6%)	719 (8%)	336 (8%)	257 (6%)	21 476 (16%)
Current smoker		37 308 (34%)	1968 (30%)	4624 (27%)	1532 (30%)	2155 (28%)	937 (22%)	2179 (26%)	1251 (29%)	1194 (29%)	43 900 (33%)
**Anthropometry and blood pressure**											
BMI, kg/m^2^		29·0 (4·7)	31·5 (5·3)	29·1 (5·0)	30·2 (5·2)	29·2 (4·9)	27·6 (4·6)	29·8 (5·0)	29·0 (4·9)	27·9 (5·1)	29·1 (4·8)
Systolic/diastolic blood pressure, mm Hg		125/82 (15/10)	133/86 (17/10)	133/85 (18/10)	131/85 (17/10)	132/85 (17/10)	137/85 (20/11)	134/85 (18/10)	133/85 (18/10)	131/84 (18/11)	127/83 (16/10)
**Antidiabetes medication**											
Any antidiabetes medication		..	..	13 553 (80%)	3610 (72%)	6341 (82%)	3602 (86%)	6425 (76%)	3681 (85%)	3447 (83%)	..
	Insulin	..	..	1176 (7%)	109 (2%)	460 (6%)	607 (15%)	399 (5%)	406 (9%)	371 (9%)	..
	Biguanide (eg, metformin)	..	..	3105 (18%)	714 (14%)	1510 (20%)	881 (21%)	1447 (17%)	855 (20%)	803 (19%)	..
	Sulfonylurea	..	..	11 660 (69%)	3225 (64%)	5532 (72%)	2903 (69%)	5543 (65%)	3162 (73%)	2955 (71%)	..
	Other antidiabetes	..	..	244 (1%)	80 (2%)	116 (2%)	48 (1%)	114 (1%)	61 (1%)	69 (2%)	..
**Other long-term medication**											
Any antihypertensive		12 048 (11%)	1030 (16%)	4780 (28%)	1222 (24%)	2081 (27%)	1477 (35%)	2784 (33%)	1123 (26%)	873 (21%)	17 858 (13%)
	Renin–angiotensin system inhibitor	8806 (8%)	793 (12%)	3782 (22%)	958 (19%)	1683 (22%)	1141 (27%)	2183 (26%)	907 (21%)	692 (17%)	13 381 (10%)
	Other antihypertensive	4321 (4%)	334 (5%)	1291 (8%)	340 (7%)	532 (7%)	419 (10%)	783 (9%)	283 (7%)	225 (5%)	5946 (4%)
Any antithrombotic		2589 (2%)	152 (2%)	357 (2%)	101 (2%)	160 (2%)	96 (2%)	222 (3%)	76 (2%)	59 (1%)	3098 (2%)
Any lipid–lowering		444 (<1%)	23 (<1%)	200 (1%)	71 (1%)	82 (1%)	47 (1%)	124 (1%)	52 (1%)	24 (1%)	667 (<1%)

Data are mean (SD) or n (%), unless otherwise stated.

* Estimated from age at recruitment and decade of diagnosis.

† Suggestive of type 1 diabetes.

‡ HbA_1c_ values greater than 9% reflect poor glycaemic control.

Participants with previously diagnosed or undiagnosed diabetes were older and less likely to be university or college educated or to be current smokers than participants without diabetes (table 1). Among those with previously diagnosed diabetes, longer time since diagnosis was associated with older age, lower BMI, and higher systolic blood pressure (table 1).

Among participants aged 35–74 years, glycaemic control in those with previously diagnosed diabetes was poor (median HbA_1c_ 8·9% [IQR 7·0–10·9]) and, despite more use of oral antidiabetes drugs and insulin in those with longer diabetes duration, glycaemic control was worse among those with longer duration (table 1). Median HbA_1c_ in the 6541 participants aged 35–74 years with undiagnosed diabetes (HbA_1c_ 7·7% [IQR 6·8–10·3]) was similar to that in the 5042 participants with previously diagnosed diabetes of duration less than 5 years (7·9% [6·4–10·3]; table 1). Median HbA_1c_ in the 110 181 participants aged 35–74 years without diabetes was 5·4% (5·2–5·7; table 1).

**Figure 1:**
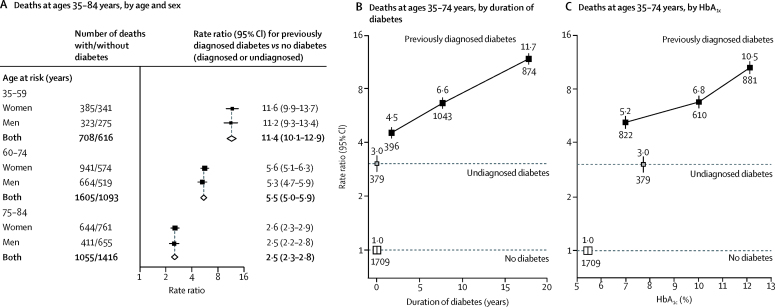
Relevance of previously diagnosed and undiagnosed diabetes to mortality from vascular, renal, or infectious causes, by age and sex (A), duration of diabetes (B), and glycaemic control (C) (A) Death rate ratios (RRs) by age and sex for deaths at ages 35–84 years, for patients with diagnosed diabetes versus those with no diabetes. Diamonds show values for men and women combined. The RRs for participants with undiagnosed diabetes (ie, no previous diagnosis but baseline HbA_1c_ ≥6·5%) compared with participants without diabetes were 4·0 (95% CI 3·3–4·9) at ages 35–59 years, 2·6 (2·3–3·0) at ages 60–74 years, and 1·4 (1·2–1·6) at ages 75–84 years, and were similar in men and women. (B) Death RRs by duration of diabetes, at ages 35–74 years. (C) Death RRs by glycaemic control, at ages 35–74 years. RRs in all panels are adjusted for age at risk, smoking status, district, educational level, height, weight, and waist and hip circumferences. In (B) and (C), RR estimates are additionally adjusted for sex, and the estimates for those with previously diagnosed diabetes are also adjusted, respectively, for any HbA_1c_ or diabetes duration differences between the groups (to the average HbA_1c_ or duration seen for all those with previously diagnosed diabetes) in such a way that their information-weighted average equals the overall RR estimate for all those with previously diagnosed diabetes versus those with no diabetes. The numbers above the squares are the RRs and the numbers below the squares are the number of deaths in that group. In all panels, the size of each square is proportional to the amount of statistical information.

During a median of 13·9 years (IQR 12·7–14·8) of follow-up among survivors, 7683 participants died at ages 35–74 years (including 4401 from vascular, renal, or infectious diseases and 318 from acute diabetic crises; table 2) and 4242 died at ages 75–84 years. For the combination of vascular disease, renal disease, or infection, the death RR comparing those with previously diagnosed diabetes with those without diagnosed or undiagnosed diabetes was larger at younger than older ages but, at a given age, was the same for men and women (figure 1A). At ages 35–74 years, the death RR for those with undiagnosed diabetes was 3·0 (95% CI 2·7–3·4); for those with previously diagnosed diabetes with a duration of less than 5 years the RR was 4·5 (4·0–5·0), for those with a duration of 5 years to less than 10 years it was 6·6 (6·1–7·1), and for those with a duration of 10 years or more it was 11·7 (10·7–12·7; figure 1B). Similarly, among participants with previously diagnosed diabetes, the death RR compared with those without diabetes was 5·2 (95% CI 4·8–5·7) for those with a baseline HbA_1c_ less than 9%, 6·8 (6·2–7·4) for those with a baseline HbA_1c_ of 9% to less than 11%, and 10·5 (9·7–11·5) for those with a baseline HbA_1c_ of 11% or higher (figure 1C). Death RRs associated with different diabetes durations and levels of glycaemic control were similar in men and women (appendix).

**Table 2:** Excess cause-specific mortality at ages 35–74 years associated with previously diagnosed or undiagnosed diabetes at recruitment

		**Number of deaths**				Death RR (95% CI) *vs* no diabetes*				**Attributable mortality**†
		No diabetes (n=110 181)	Undiagnosed diabetes (n=6541)	Previously diagnosed diabetes		No diabetes	Undiagnosed diabetes	Previously diagnosed diabetes		
				HbA_1c_ <9% (n=8475)	HbA_1c_ ≥9% (n=8465)			HbA_1c_ <9%	HbA_1c_ ≥9%	
All-cause mortality		3944	595	1184	1960	1·0	2·1 (1·9–2·2)	3·0 (2·8–3·3)	5·2 (4·9–5·5)	35%
Any vascular, renal, or infectious		1709	379	822	1491	1·0	3·0 (2·7–3·4)	4·9 (4·5–5·3)	9·1 (8·5–9·8)	51%
	Renal	224	115	301	679	1·0	8·0 (6·4–10·1)	15·8 (13·2–18·9)	33·6 (28·8–39·3)	79%
	Cardiac	697	127	244	362	1·0	2·4 (2·0–2·9)	3·3 (2·8–3·8)	5·3 (4·7–6·0)	37%
	Infectious	434	86	166	266	1·0	2·8 (2·2–3·5)	4·0 (3·3–4·8)	6·6 (5·6–7·7)	43%
	Cerebrovascular	268	37	79	132	1·0	1·7 (1·2–2·4)	2·6 (2·0–3·3)	4·7 (3·8–5·8)	32%
	Other vascular	86	14	32	52	1·0	2·1 (1·2–3·8)	3·5 (2·3–5·3)	6·3 (4·4–9·0)	40%
Acute diabetic crises‡		48	28	86	156	..	..	..	..	100%
Neoplastic		1051	85	102	121	1·0	1·1 (0·9–1·4)	1·0 (0·8–1·2)	1·2 (1·0–1·5)	2%
Cirrhotic		436	42	58	55	1·0	1·2 (0·9–1·7)	1·4 (1·0–1·8)	1·3 (1·0–1·8)	6%
Chronic obstructive pulmonary disease		136	12	27	20	1·0	0·9 (0·5–1·7)	1·5 (1·0–2·3)	1·2 (0·8–2·0)	6%
Ill-defined, other, or external		564	49	89	117	1·0	1·2 (0·9–1·7)	1·7 (1·4–2·2)	2·3 (1·9–2·8)	14%

RR=rate ratio.

* Death RR estimates for those with versus without diabetes at recruitment were adjusted for age, sex, district, educational level, smoking status, and anthropometric measures.

† For each of previously diagnosed diabetes with HbA_1c_ ≥9%, previously diagnosed diabetes with HbA_1c_ <9%, and undiagnosed diabetes, the number of deaths attributable to the excess risk associated with that level of diabetes was calculated as number of deaths × (RR–1)/RR, where RR is the cause-specific death RR for that group relative to those without diabetes. These three numbers were then summed and presented as a percentage of all such deaths. For example, for renal death, the calculation was 100 × ([679 × 32·6/33·6] + [301 × 14·8/15·8] + [115 × 7·0/8·0])/1319=79%.

‡ Death RR estimates are not shown for deaths attributed to acute diabetic crises as all such deaths were due to diabetes, irrespective of whether diabetes was diagnosed before recruitment.

Diabetes was not strongly associated with death from the combination of all other causes apart from acute diabetic crises (appendix). For all-cause mortality, the death RR at ages 35–74 years was 2·1 (95% CI 1·9–2·2) for those with undiagnosed diabetes and 4·1 (3·9–4·3) for those with previously diagnosed diabetes. When previously diagnosed diabetes was broken down by duration, participants with a duration of less than 5 years had an all-cause mortality RR of 2·9 (2·7–3·2), those with a duration of 5 years to less than 10 years had an RR of 3·9 (3·7–4·2), and those with a duration of 10 years or more had an RR of 6·4 (6·0–6·9). When broken down by glycaemic control, participants with a baseline HbA_1c_ of less than 9% had an all-cause mortality RR of 3·2 (3·0–3·5), those with a baseline HbA_1c_ of 9% to less than 11% had an RR of 4·0 (3·8–4·4), and those with a baseline HbA_1c_ of 11% or higher had an RR of 5·9 (5·5–6·3; appendix).

**Figure 2:**
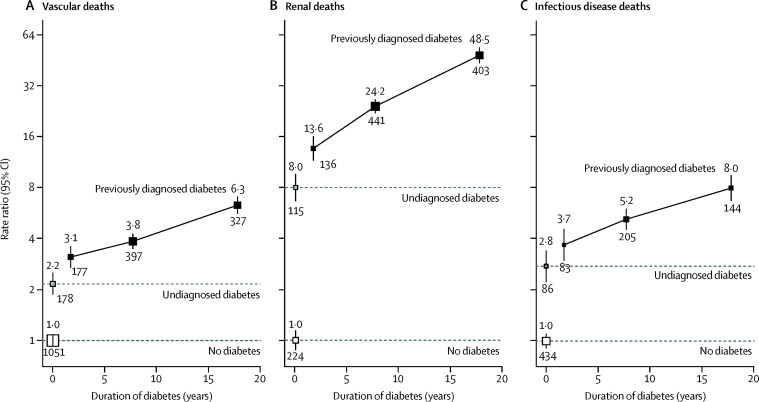
Relevance of previously diagnosed and undiagnosed diabetes to mortality from vascular (A), renal (B), and infectious (C) causes at ages 35–74 years, by duration of diabetes Rate ratios (RRs) are adjusted for age at risk, sex, smoking status, district, educational level, height, weight, and waist and hip circumferences, as well as HbA_1c_ for participants with previously diagnosed diabetes. The numbers above the squares are the RRs and the numbers below the squares are the number of deaths in that group. In all panels, the size of each square is proportional to the amount of statistical information.

**Figure 3:**
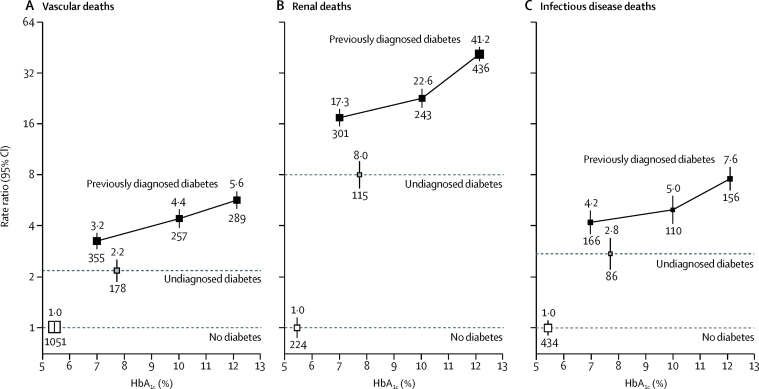
Relevance of previously diagnosed and undiagnosed diabetes to mortality from vascular (A), renal (B), and infectious (C) causes at ages 35–74 years, by glycaemic control Rate ratios (RRs) are adjusted for age at risk, sex, smoking status, district, educational level, height, weight, and waist and hip circumferences, as well as diabetes duration for participants with previously diagnosed diabetes. The numbers above the squares are the RRs and the numbers below the squares are the number of deaths in that group. In all panels, the size of each square is proportional to the amount of statistical information.

Diabetes was particularly strongly related to death from renal disease (Figure 2, Figure 3). For example, for those previously diagnosed with diabetes with a duration of 5 years to less than 10 years or baseline HbA_1c_ 9% to less than 11%, the death RRs versus those without diabetes were 3·8 (3·4–4·3) or 4·4 (3·8–5·0), respectively, for vascular disease, but 24·2 (20·5–28·6) or 22·6 (18·8–27·2), respectively, for renal disease. For each of vascular disease, renal disease, and infection, separately and in combination, the increase in the death RR seen with longer duration of diabetes was generally steeper for deaths at ages 35–59 years than deaths at ages 60–74 years (appendix); for glycaemic control, the increase in RRs seen with increasing HbA_1c_ was broadly similar in these two age ranges (appendix). For the combination of death from vascular, renal, or infectious causes, the overall diabetes death RR at ages 35–74 years was 7·0 (95% CI 6·7–7·3), and was similar irrespective of sex, residential district, and smoking status, and broadly similar irrespective of education, but significantly larger in those in the lowest versus highest quarter of the baseline BMI distribution, perhaps reflecting the worse glycaemic control in those with diabetes with low BMI (appendix). Among the participants with previously diagnosed diabetes, those with worse glycaemic control (HbA_1c_ ≥9%) at a given diabetes duration had, on average, about twice the death rate from these causes than those with better glycaemic control (HbA_1c_ <9%); and, at a given HbA_1c_, those with longer diabetes duration (≥10 years) had, on average, about twice the death rate of those with shorter duration (<10 years; appendix). Among participants without diabetes, higher HbA_1c_ was not positively related to mortality (appendix).

Overall, between the ages of 35 and 74 years, the excess risk of death associated with previously diagnosed or undiagnosed diabetes accounted for 35% of all deaths (the sum of 4% associated with undiagnosed diabetes, 10% associated with previously diagnosed diabetes with HbA_1c_ <9%, and 21% associated with previously diagnosed diabetes with HbA_1c_ ≥9%). This excess risk included half of the deaths from a vascular, renal, or infectious cause (including four-fifths of renal deaths) and less than a tenth of deaths from the combination of all other causes apart from acute diabetic crises (table 2).

When standardised to national death rates for Mexico for the year 2012, the absolute excess risk of death associated with diabetes (undiagnosed, previously diagnosed with HbA_1c_ <9%, or previously diagnosed with HbA_1c_ ≥9%) was greatest for renal disease (appendix). The main analyses of mortality were not materially affected by additional adjustments for alcohol intake, physical activity, and blood pressure; by inclusion of data from participants who had a previously diagnosed disease other than diabetes; or by imputation of data for participants with missing covariate data (data not shown).

## Discussion

In Mexico, diabetes is strongly associated with deaths from vascular disease, renal disease, and infection,^8^ with risks increasing substantially both with longer diabetes duration and worse glycaemic control. Compared with those without diabetes (ie, those not diagnosed and with HbA_1c_ <6·5%) at recruitment, death rates from vascular, renal, and infectious causes combined were about three times higher among those with HbA_1c_-defined undiagnosed diabetes, five times higher in those with diagnosed diabetes with a duration less than 5 years, seven times higher in those with diagnosed diabetes with a duration of 5 to less than 10 years, and 12 times higher in those with diagnosed diabetes with a duration of 10 years or more. Poorer glycaemic control was associated with even higher death RRs; at any given duration of diabetes, an HbA_1c_ of 9% or more was associated with about a doubling in the death rate compared with an HbA_1c_ of less than 9%.

Our results showed that longer duration of diabetes was associated with a more steeply increased risk of renal death than from vascular disease or infection. This finding is consistent with observations from diabetes populations from high-income countries, in which microvascular complications (such as diabetic kidney disease) were more steeply associated with duration of diabetes than were macrovascular complications.^4^ However, our estimate of the rate at which the RR for renal death increased with longer duration of diabetes (about a 50% further increase in the RR per 5 years longer duration) was somewhat larger than that seen in earlier studies, perhaps reflecting the worse glycaemic control or low use of renin–angiotensin system blockade, or both,^14^ in this Mexican population; whereas our estimate of the rate at which the RRs for vascular death increased with longer duration of diabetes (about a 20% further increase in the RR per 5 years longer duration) was similar to that seen previously.^4^, ^5^, ^6^ In contemporaneous data from China, the relation between duration of diabetes and mortality has been shown to be steeper in rural than in urban areas, perhaps also reflecting regional differences in the availability of medical care.^15^ Previous studies have also shown strong associations between diabetes and deaths from infection,^2^, ^15^ but the effect of duration of diabetes on infectious mortality is not well documented. For fatal infections in our study, the rate at which death RRs increased was similar to the finding for vascular mortality (about 20–30% further increase per 5 years longer diabetes duration).

Prospective studies of mostly high-income countries have found that death RRs associated with diabetes are somewhat higher for women than men, particularly for vascular disease,^2^, ^3^, ^16^ and that longer duration of diabetes might be more strongly associated with cardiac mortality in women than in men.^17^ Contrary to these observations, our study of Mexican adults found similar death RRs for diabetes in men and women, and similar rates of increase in these RRs with duration of diabetes. Investigators of a study in Brazil also reported diabetes death RRs that are higher than those reported in high-income countries and that did not differ between women and men.^18^

For a given duration of diabetes, an HbA_1c_ of 9% or higher was associated with about a doubling in the risk of vascular, renal, or infectious disease-related death compared with an HbA_1c_ of less than 9%. The difference in median HbA_1c_ between these two groups was about 4% (10·9% *vs* 7·0%). Large-scale trials of intensive versus standard glycaemic control managed to achieve a 0·9% difference in HbA_1c_ (7·7% *vs* 6·8%), which, over about 5 years, translated to a reduction in risk of myocardial infarction of 15%, a reduction in risk of any major cardiovascular event of about 10%,^19^ and a reduction in markers of progressive kidney disease of 20%.^20^ Our estimates of the difference in death rates between those with diabetes and an HbA_1c_ of less than 9% compared with those with diabetes and an HbA_1c_ of 9% or higher are broadly consistent with what might be predicted from these trials, because the relation in our study between HbA_1c_ greater than about 7% and vascular deaths seemed to be continuous and log-linear (figure 3A).

By contrast, the relation between HbA_1c_ more than about 7% and renal mortality in our study seemed to steepen progressively with higher HbA_1c_ (figure 3B). The shape of the positive association between HbA_1c_ and death from infection was less clear (figure 3C), but might also be curvilinear. If these associations are causal, then even a modest reduction in HbA_1c_ among those with the worst glycaemic control might substantially reduce the risk of death from renal and infectious diseases.

Consistent with findings from a large-scale meta-analysis of cohorts,^21^ we found evidence of increased risk among participants with undiagnosed diabetes (median baseline HbA_1c_ 7·7%), but no evidence that HbA_1c_ less than 6·5% was positively related to death from any particular cause (appendix).

Previous meta-analyses have included information about non-fatal cardiovascular outcomes, and a limitation of the present study is the absence of such information for non-fatal diseases. We were also unable to account for any effects of diabetes incidence or improvements in diabetes care during the period studied (including increased use of blood pressure-lowering and cholesterol-lowering drugs),^7^ but this would, if anything, result in our death RRs being underestimates. Other relevant biomarkers (such as blood lipids and markers of renal function) have not yet been measured, so we are not yet able to explore their role as potential effect mediators or confounders. Also, few participants in our study had diabetes indicative of type 1 diabetes (table 1), meaning that our results are only really relevant to type 2 diabetes. Although our results were generated from just two districts of Mexico City, previous national surveys from a similar time period found that glycaemic control was, if anything, worse than that seen in our study.^7^, ^8^ Finally, we are unable to rule out the possibility of some residual confounding.

In 2016, WHO recognised the enormous scale of the worldwide diabetes problem and published its first global report on diabetes.^22^ This document recommended that in addition to strengthening the health system response at a primary care level and making insulin available and affordable to those that need it, an effort should also be made to prioritise actions to prevent people becoming overweight and obese, beginning in early childhood. A coordinated, multicomponent intervention encouraging consumption of healthy foods, discouraging the consumption of sugary food and drink, and a supportive environment for physical activity are recommended. The Mexican National Strategy for Overweight, Obesity and Diabetes was announced in 2013. It included health education, improved opportunities for exercise, taxation of sugary drinks and high-calorie foods, and earlier identification and monitoring of major health risk factors, including diabetes.^23^ Since recruitment in our study ended in 2004, the prevalence of diabetes in Mexico has continued to rise.^7^ In 2016, Mexico declared the epidemic of diabetes a national emergency, with the aim of improving the quality of care for people with the disease, ensuring that, irrespective of socioeconomic status, people have access to medicines, are regularly screened for complications, and that any complications are treated promptly.^24^ The effect of these and other recent policies^25^, ^26^ on the incidence of type 2 diabetes and outcomes for people with diabetes will not be known for some years, but our study suggests that major health benefits on a wide range of causes of premature death would be expected if the development of diabetes can be delayed and effective glycaemic control can be implemented among those who already have the disease.

In conclusion, in this study of Mexican adults, vascular, renal, and infectious disease death rates increased steeply with both duration of diabetes and with worse glycaemic control. In populations in which obesity and diabetes are overwhelming health services, the onset of diabetes needs to be delayed—and its treatment improved—to substantially reduce premature adult mortality.
