# Supplementary material for: Effect of diabetes duration and glycaemic control on 14-year cause-specific mortality in Mexican adults: a blood-based prospective cohort study
Source: Lancet Diabetes Endocrinol. 2018 Jun;6(6):455–63. doi: 10.1016/S2213-8587(18)30050-0 (PMC5966587; doi:10.1016/S2213-8587(18)30050-0)
Supplement: Supplementary appendix [file mmc1.pdf]

THE LANCET
Diabetes & Endocrinology

Supplementary appendix

This appendix formed part of the original submission and has been peer reviewed. We post it as supplied by the authors.

Supplement to: Herrington W G, Alegre-Díaz J, Wade R, et al. Effect of diabetes duration and glycaemic control on 14-year cause-specific mortality in Mexican adults: a blood-based prospective cohort study. *Lancet Diabetes Endocrinol* 2018; published online March 19. http://dx.doi.org/10.1016/S2213-8587(18)30050-0.

Effect of diabetes duration and glycaemic control on 14-year cause-specific mortality in Mexican adults: a blood-based prospective cohort study

Supplementary Material, Table of Contents

**Supplementary figures**	**Page**
Sex-specific relevance of previously-diagnosed and, separately, undiagnosed diabetes to mortality at ages 35-74 years from a vascular, renal or infectious cause, by A) duration of diabetes; and B) glycaemic control	3
Age-, sex-, diabetes-duration- and glycaemic-control-specific relevance of previously-diagnosed and, separately, undiagnosed diabetes to:	4-5
‘Other’ (i.e., not vascular, renal or infectious) causes of death except acute diabetic crises	
All-cause mortality	
Relevance of previously-diagnosed diabetes to mortality at ages 35-74 years from vascular, renal and infectious causes:	6-9
by duration of diabetes and age at risk	
by glycaemic control and age at risk	
by other characteristics	
by duration of diabetes and glycaemic control	
Relevance of baseline HbA1c to cause-specific mortality at ages 35-84 among participants with no diagnosed or undiagnosed diabetes at recruitment	10
Mortality rates at ages 35-74 years for each cause of death among participants with previously-diagnosed diabetes, undiagnosed diabetes and no diabetes at recruitment	11

**Supplementary Tables**	
Number of vascular, renal and infectious deaths at ages 35-74 years by underlying cause (ICD-10 code), among 133 662 participants with no other chronic disease (other than diabetes) at recruitment	12
Death rate ratios associated with previously diagnosed and undiagnosed diabetes for mortality from vascular, renal, and infectious causes (separately and combined) at ages 35–74 years, by A) duration of diabetes and B) HbA1c - Comparison of ‘conventional’ 95% confidence intervals with ‘group-specific’ 95% confidence intervals	13
Baseline characteristics among men and women aged 75-84 without chronic disease at recruitment (other than diabetes)	14

Webfigure 1: Sex-specific relevance of previously-diagnosed and, separately, undiagnosed diabetes to mortality at ages 35-74 years from a VASCULAR, RENAL or INFECTIOUS cause by A) duration of diabetes; and B) glycaemic control

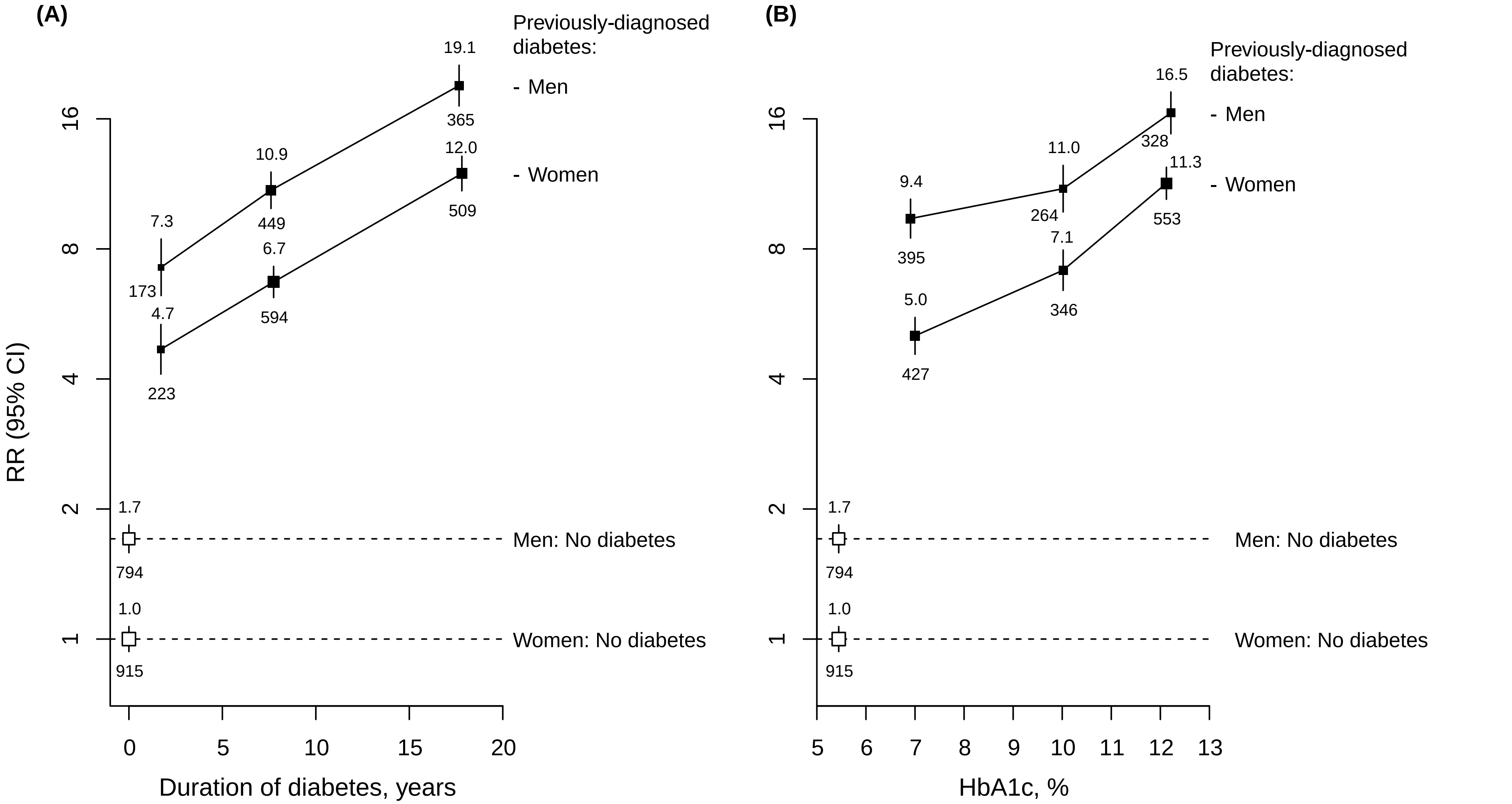

Rate ratios exclude data from any participants who had previously diagnosed chronic disease other than diabetes (chronic kidney disease, ischaemic heart disease, stroke, cirrhosis, cancer or emphysema) and are adjusted for age at risk, smoking status, district, educational level, height, weight, and waist and hip circumferences. In addition, the rate ratio estimates for men and women with previously-diagnosed diabetes (black filled squares) are also adjusted for the effect on risk of any A) HbA1c differences or B) diabetes duration differences between those groups. The 95% confidence intervals reflect the variance of the log risk in that 1 group, taking into account the variance of the log risk in the reference group without diabetes. The rate ratio and confidence interval estimates for those with undiagnosed diabetes are not plotted, but would be shown at 3.0 (2.6-3.5) for women with undiagnosed diabetes and 5.3 (4.5-6.1) for men with undiagnosed diabetes. In each panel, the vertical distance between the two horizontal dashed lines is about the same as the vertical distance between the two solid lines, demonstrating that the relative risk for those with previously-diagnosed diabetes versus no diabetes was about the same for men and women, and that this was true irrespective of diabetes duration or glycaemic control.

Webfigure 2: Relevance of previously-diagnosed and, separately, undiagnosed diabetes to mortality from OTHER causes except acute diabetic crises by: A) age and sex; B) duration of diabetes; and C) glycaemic control

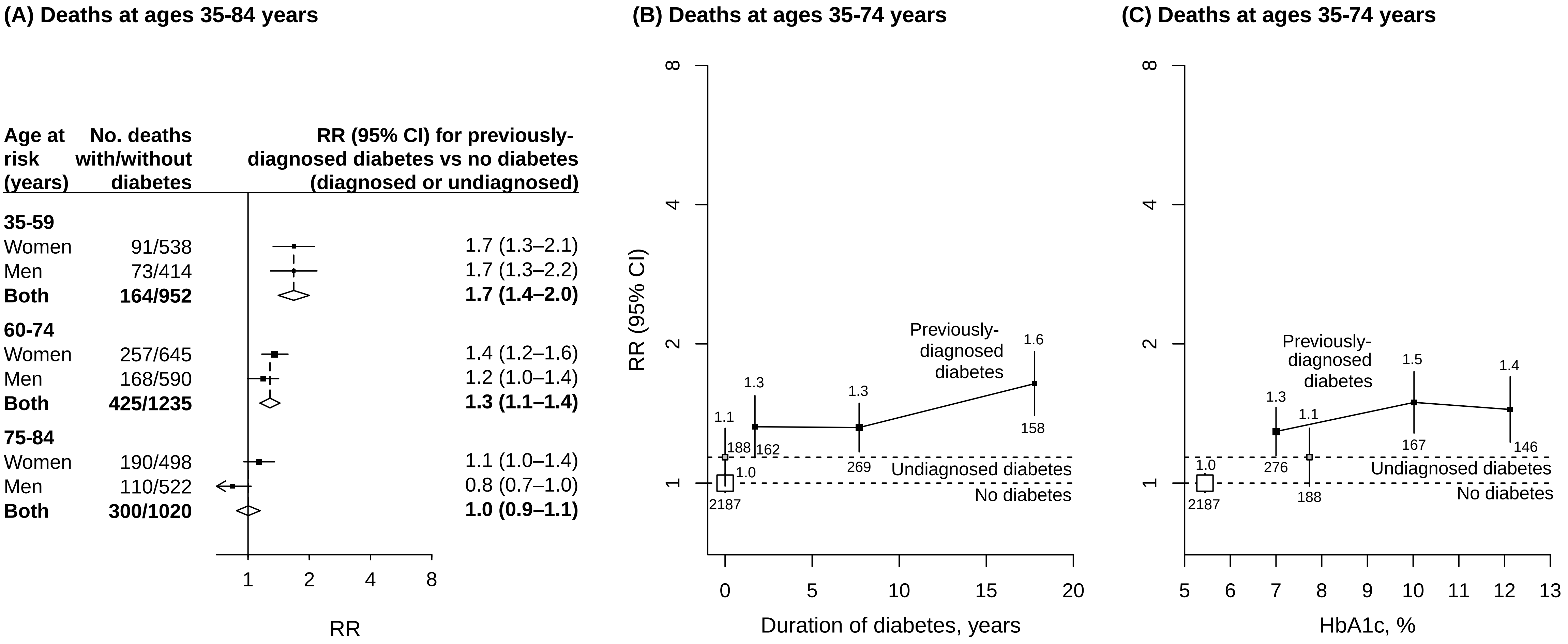

See Figure 1 for details of exclusions, analyses and conventions. The rate ratios for participants with undiagnosed diabetes (ie, no previous diagnosis but baseline HbA1c ≥ 6.5%) compared with participants without diabetes were 1.5 (1.2-1.9) at ages 35-59, 1.0 (0.8-1.2) at ages 60- 74 and 1.2 (1.0-1.4) at ages 75-84, and were similar in men and women.

Webfigure 3: Relevance of previously-diagnosed and, separately, undiagnosed diabetes to ALL-CAUSE mortality by: A) age and sex; B) duration of diabetes; and C) glycaemic control

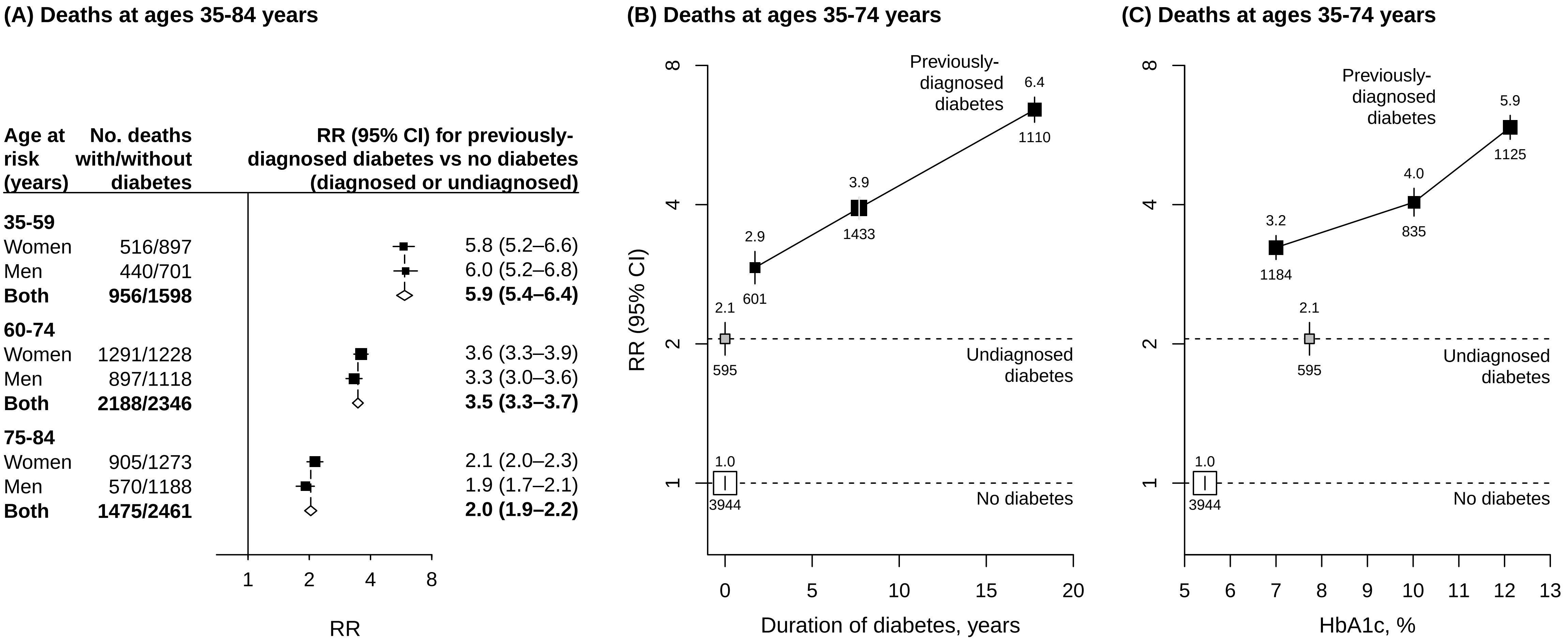

See Figure 1 for details of exclusions, analyses and conventions. The rate ratios for participants with undiagnosed diabetes (ie, no previous diagnosis but baseline HbA1c ≥ 6.5%) compared with participants without diabetes were 2.5 (2.2-2.9) at ages 35-59, 1.8 (1.6-2.0) at ages 60-74 and 1.4 (1.2-1.5) at ages 75-84, and were similar in men and women.

Webfigure 4: Relevance of previously-diagnosed diabetes to mortality at ages 35-74 years from VASCULAR, RENAL and INFECTIOUS causes, by duration of diabetes and age at risk

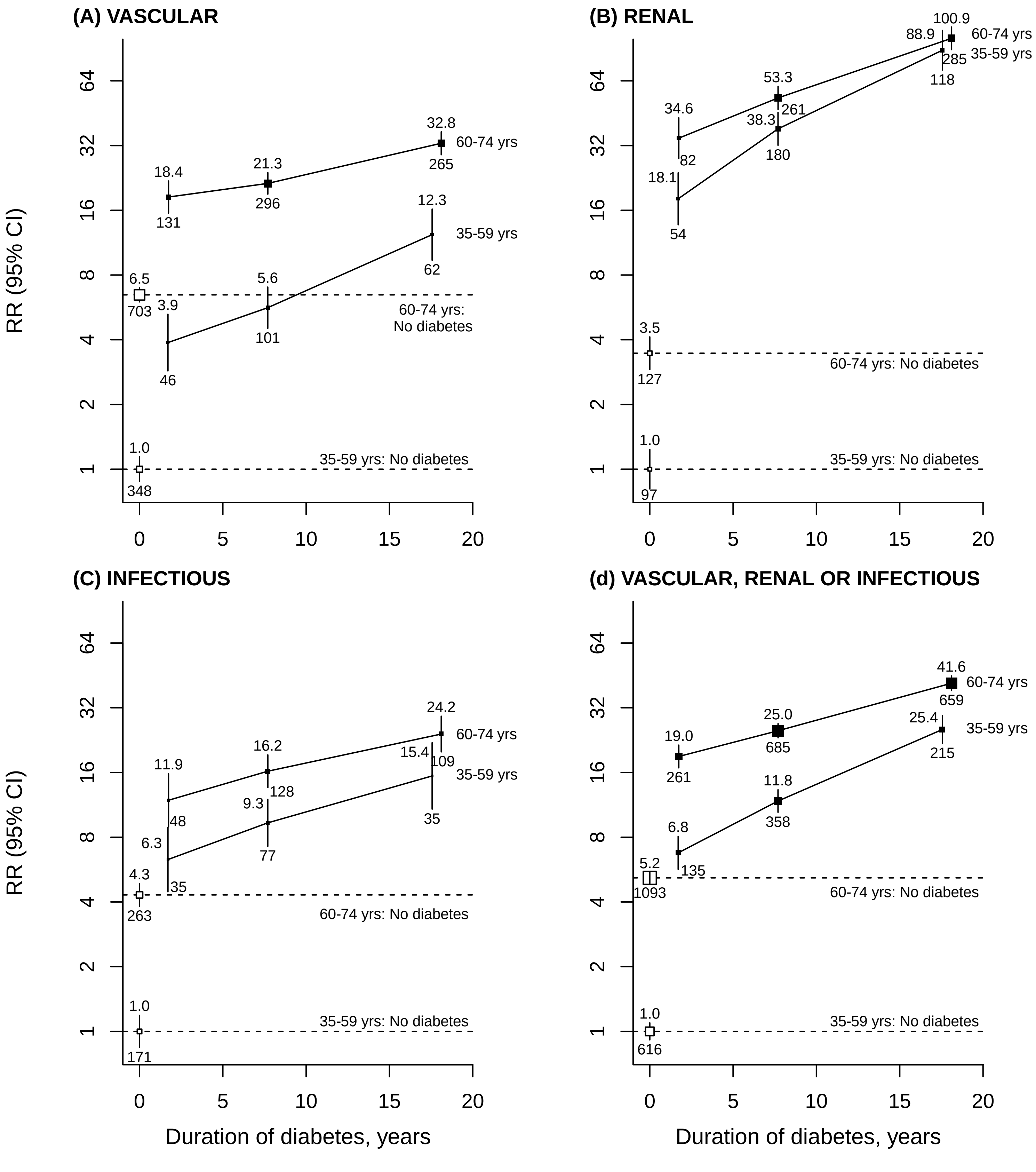

Exclusions, analyses and conventions as for panel B of Figure 1, with the exception that rate ratios are now shown separately for deaths at ages 35-59 and deaths at ages 60-74. The age- specific death rate ratios and CIs corresponding to undiagnosed diabetes are not shown in this plot, but the average RRs at ages 35-74 are given in panel B of figure 1 and panels A-C of figure 2.

Webfigure 5: Relevance of previously-diagnosed diabetes to mortality at ages 35-74 years from VASCULAR, RENAL and INFECTIOUS causes, by glycaemic control and age at risk

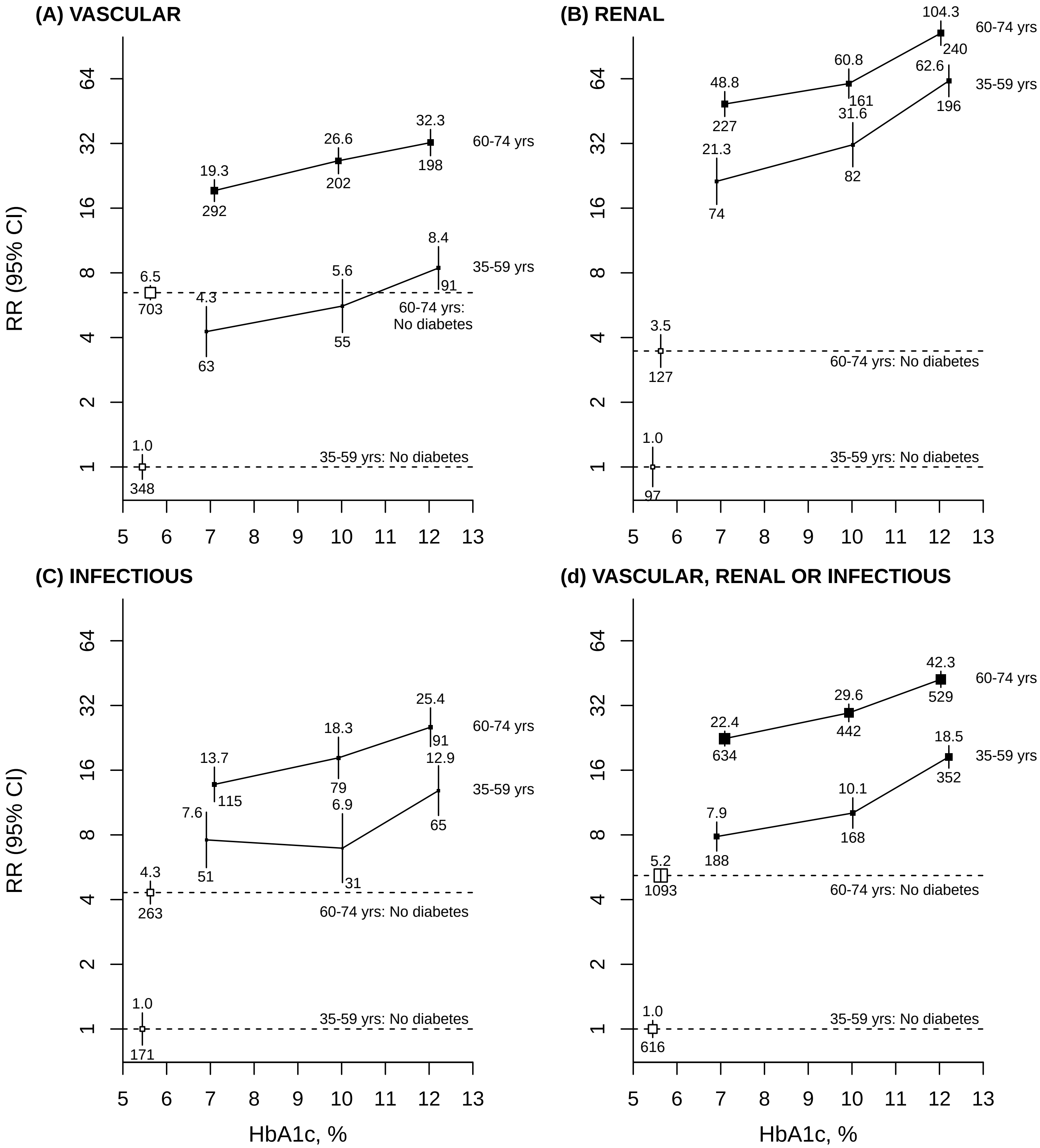

Exclusions, analyses and conventions as for panel C of Figure 1, with the exception that rate ratios are now shown separately for deaths at ages 35-59 and deaths at ages 60-74. The age- specific death rate ratios and CIs corresponding to undiagnosed diabetes are not shown in this plot, but the average RRs at ages 35-74 are given in panel C of figure 1 and panels A-C of figure 3.

Webfigure 6: Relevance of previously-diagnosed diabetes to mortality at ages 35-74 years from a VASCULAR, RENAL or INFECTIOUS cause, by particular characteristics

Variable	Median HbA1c with/without diabetes, %	No. deaths with/without diabetes	Death RR (95% CI) for previously-diagnosed diabetes vs no diabetes
Age			
35−59	9.5/5.4	708/616	11.5 (10.0–13.1)
60−74	8.4/5.6	1605/1093	5.5 (5.1–5.9)
Sex			
Women	9.0/5.4	1326/915	7.2 (6.6–7.9)
Men	8.8/5.4	987/794	6.7 (6.1–7.4)
District			
Coyoácan	8.8/5.4	694/618	7.2 (6.5–8.1)
Iztapalapa	9.0/5.4	1619/1091	6.8 (6.3–7.4)
Smoking			
Never	8.9/5.4	1062/726	7.1 (6.4–7.8)
Former	8.7/5.4	544/357	6.8 (5.9–7.8)
Current	9.2/5.4	707/626	6.9 (6.2–7.8)
Education			
University	8.6/5.4	147/194	8.0 (6.4–9.9)
High school	9.0/5.4	358/370	8.1 (6.9–9.3)
Elementary	9.0/5.5	1440/906	7.1 (6.5–7.7)
Other	8.9/5.5	368/239	5.0 (4.2–5.9)
BMI (kg/m^2)			
I (23.9)	9.7/5.4	829/404	10.7 (9.5–12.1)
II	9.2/5.4	570/347	8.9 (7.8–10.2)
III	8.6/5.4	446/405	5.9 (5.2–6.8)
IV (34.5)	8.3/5.6	468/553	4.5 (3.9–5.1)
**Overall**		**2313/1709**	**7.0 (6.7–7.3)**

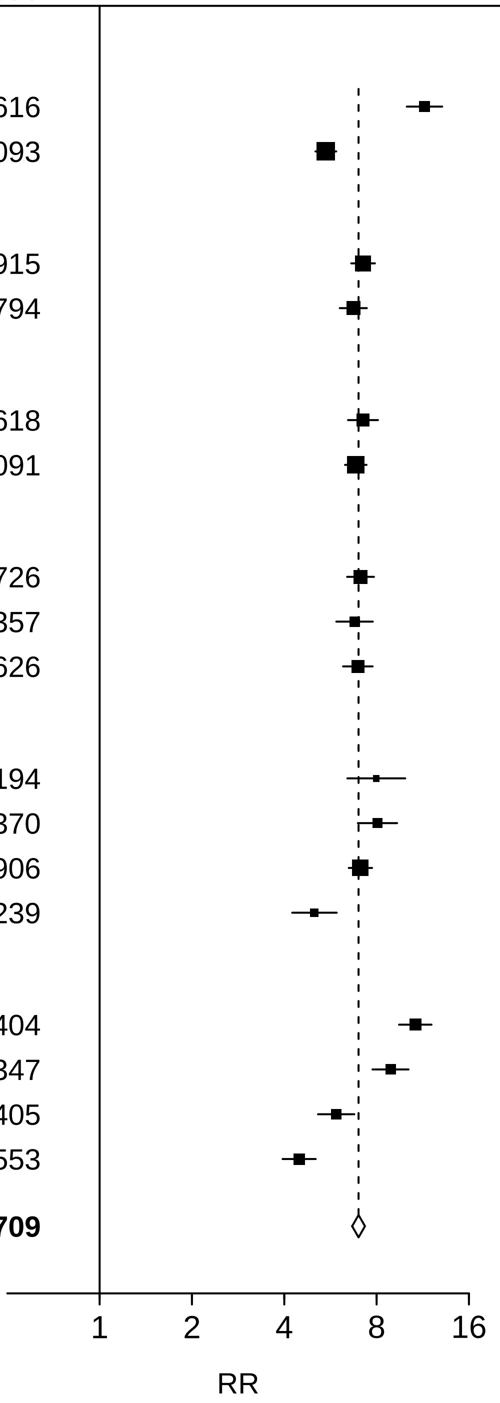

Rate ratios exclude data from any participants who had previously-diagnosed chronic disease other than diabetes (chronic kidney disease, ischaemic heart disease, stroke, cirrhosis, cancer, or emphysema) and are adjusted for or, where appropriate, stratified by, age at risk, sex, district, smoking status, educational level and body mass index (split into 4 equally-sized groups). Rate ratios are provided with their 95% confidence intervals and compare participants with previously-diagnosed diabetes with participants with no previous diagnosis and baseline HbA1c<6.5%. The rate ratios corresponding to those with undiagnosed diabetes (ie, no previous diagnosis but baseline HbA1c ≥ 6.5%) are not shown. The size of each square is proportional to the amount of data available and the line through it represents its 95% confidence interval.

Webfigure 7: Relevance of previously-diagnosed and, separately, undiagnosed diabetes to mortality at ages 35-74 years from a VASCULAR, RENAL or INFECTIOUS cause, by duration of diabetes and glycaemic control

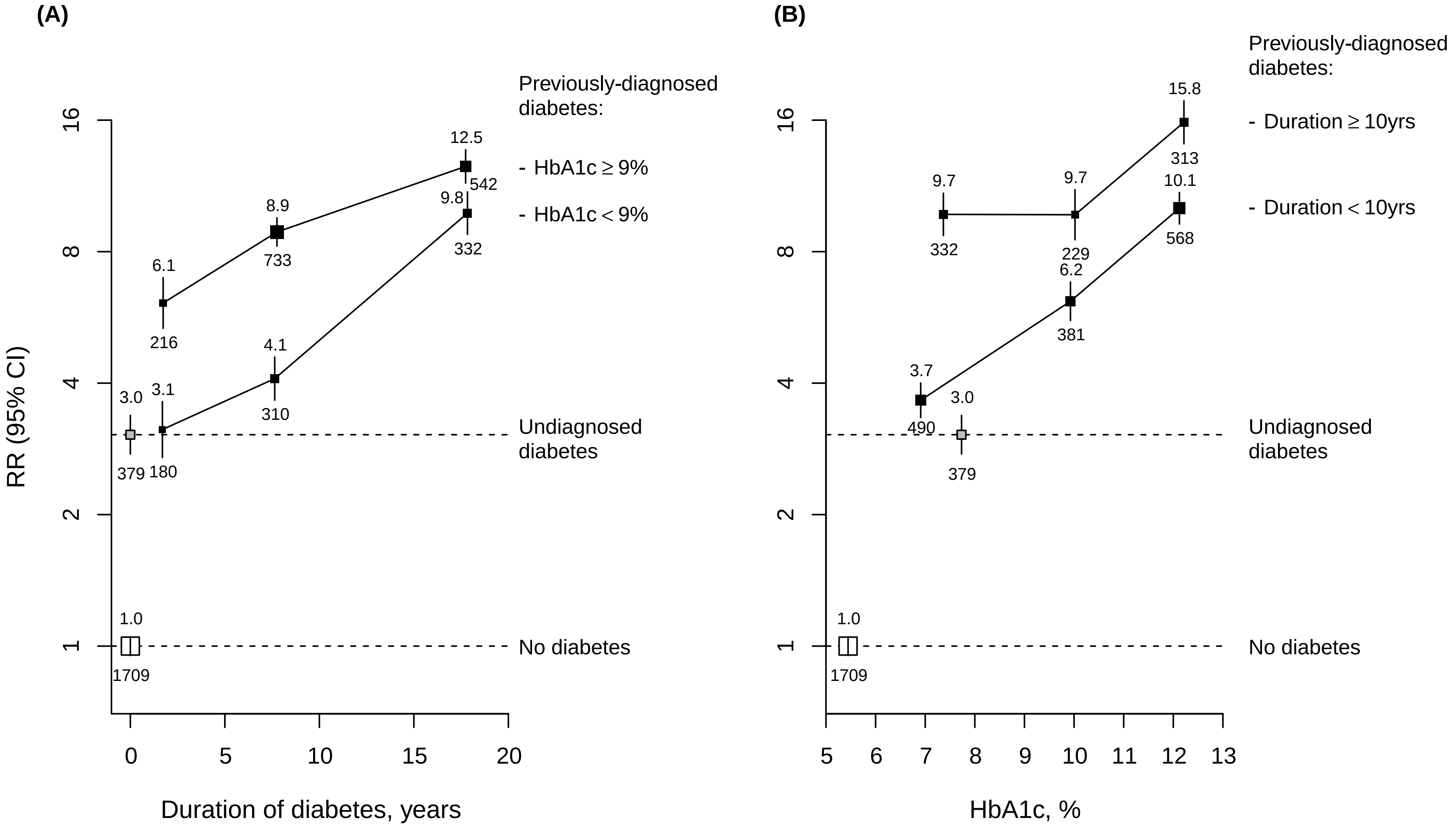

Exclusions, analyses and conventions as for panels B and C of Figure 1, with the exception that, for panel A, each of the three groups of participants with previously-diagnosed diabetes (of duration <5 years, ≥ 5 to <10 years and ≥ 10 years) are now stratified by, instead of adjusted for, their baseline HbA1c (<9% versus ≥ 9%), and, for panel B, each of the three groups of participants with previously- diagnosed diabetes (with HbA1c <9%, ≥ 9 to <11% and ≥ 11%) are now stratified by instead of adjusted for their duration of diabetes (<10 versus ≥ 10 years).

Webfigure 8: Relevance of baseline HbA1c to cause-specific mortality at ages 35-84 among participants with no diagnosed or undiagnosed diabetes at recruitment

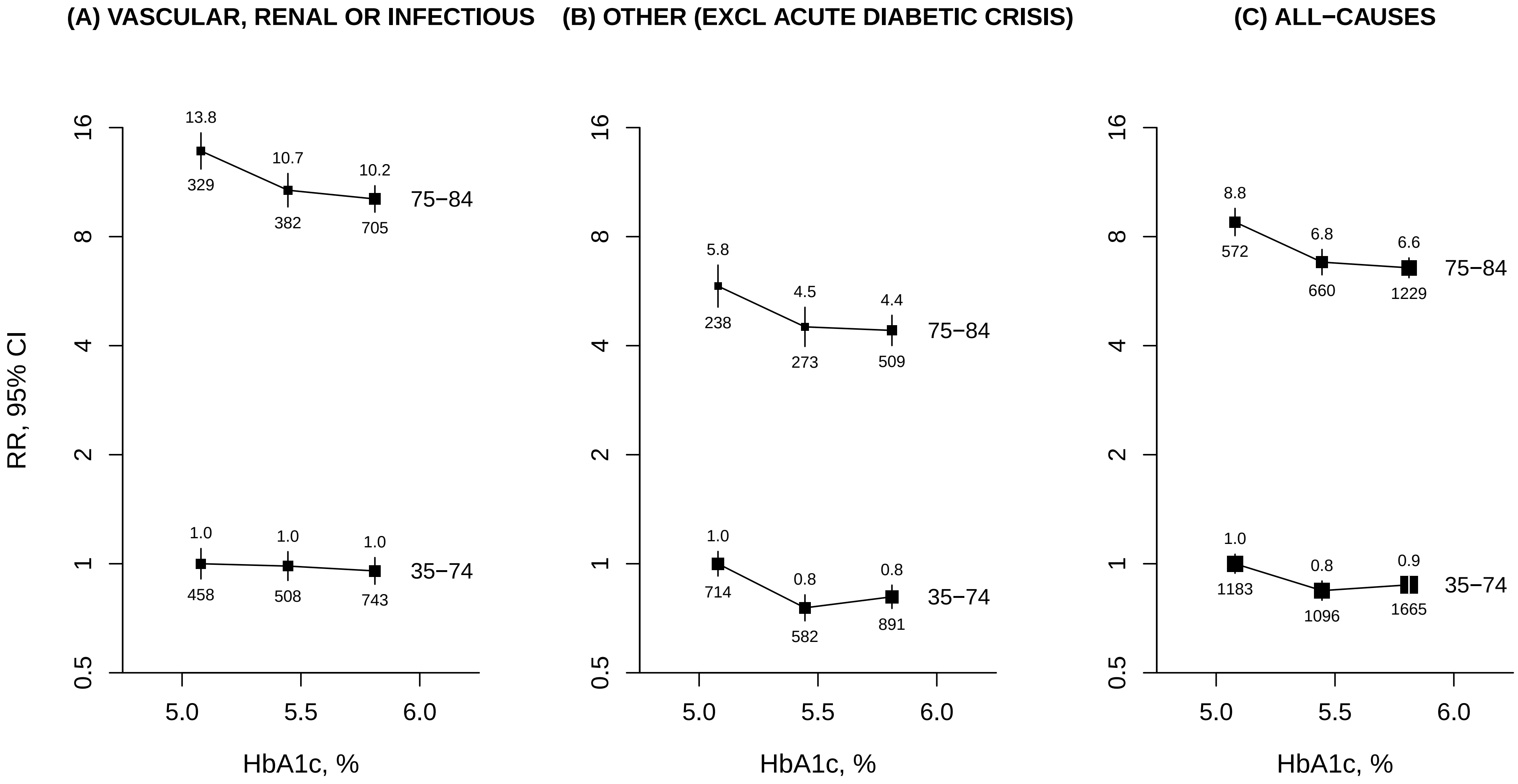

Rate ratios exclude data from participants with previously-diagnosed or undiagnosed diabetes, or with any other previously-diagnosed chronic disease (chronic kidney disease, ischaemic heart disease, stroke, cirrhosis, cancer or emphysema). Analyses are adjusted for age at risk, sex, district, smoking status, educational level, height, weight, and waist and hip circumferences. For each age group, rate ratios are shown for three groups defined by the baseline HbA1c distribution of all aged 35-84 at recruitment without diabetes. The size of each square is proportional to the amount of data available and the 95% confidence interval through it reflects the variance of the log risk in that 1 group, taking into account the variance of the log risk in the (arbitrary) reference group assigned a rate ratio of 1.00.

Webfigure 9: Mortality rates at ages 35-74 years for each cause of death among participants with previously-diagnosed diabetes, undiagnosed diabetes and no diabetes at recruitment

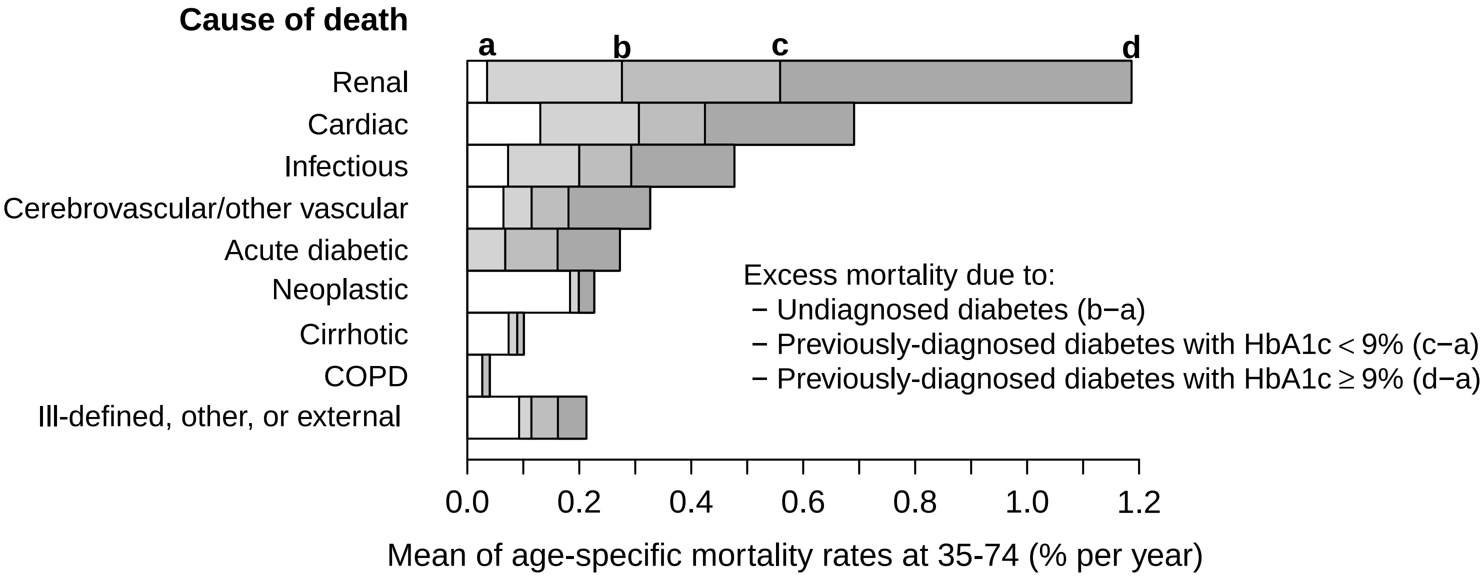

Shown are the absolute estimated disease-specific rates of death for those with previously-diagnosed diabetes and HbA1c at recruitment ≥ 9%, previously-diagnosed diabetes and HbA1c at recruitment<9%, undiagnosed diabetes at recruitment, and no diabetes at recruitment. The analysis combines the percentages of participants in the current study who died between 35 and 74 years of age from particular diseases, the disease-specific rate ratios for death at 35 to 74 years of age, and 2012 national mortality rates in Mexico. The rates for all bars sum to the rates of death from any cause. The unshaded portions of the bars represent the mortality rate for the specific cause of death among participants without diabetes at recruitment. For acute diabetic crises, the few deaths among those without diabetes at recruitment are attributed to the undiagnosed diabetes bar. The weighted average of the death rates shown (for 8% of persons with previously−diagnosed diabetes and HbA1c ≥ 9%, 8% persons with previously-diagnosed diabetes and HbA1c<9%, 5% of persons with undiagnosed diabetes and 79% of persons without diabetes) match uniformly age-standardised 2012 Mexican national rates at ages 35 to 74 years of age for 50% men plus 50% women. Infectious diseases include peptic ulcer disease and exclude any infection in another plotted category.

Webtable 1: Number of vascular, renal and infectious deaths at ages 35-74 years by underlying cause (ICD-10 code), among 133 662 participants with no other chronic disease (other than diabetes) at recruitment

Underlying cause of death	ICD-10 codes (number of such deaths recorded by 1.1.2016)
**Cardiac (1430 deaths)**	I050 (1), I051 (1), I059 (12), I070 (1), I071 (1), I080 (2), I081 (1), I091 (1), I099 (12), I110 (56), I119 (6), I200 (4), I209 (1), I210 (15), I211 (7), I219 (1052), I221 (1), I249 (6), I251 (28), I252 (1), I255 (1), I258 (4), I259 (53), I270 (5), I272 (1), I279 (5), I301 (1), I319 (1), I330 (4), I340 (2), I348 (1), I350 (8), I351 (1), I358 (1), I38X (4), I420 (7), I442 (3), I443 (1), I460 (1), I469 (6), I471 (1), I472 (2), I489 (1), I48X (5), I490 (4), I500 (29), I501 (7), I509 (42), I515 (1), I518 (2), I519 (9), Q210 (1), Q231 (1), R570 (6)
**Cerebrovascular (516 deaths)**	I600 (1), I602 (1), I608 (1), I609 (52), I61 (1), I610 (1), I614 (1), I615 (2), I618 (1), I619 (166), I620 (6), I629 (3), I633 (2), I634 (10), I635 (2), I638 (2), I639 (42), I649 (1), I64X (65), I669 (5), I671 (5), I672 (1), I673 (1), I674 (2), I678 (42), I679 (81), I693 (4), I694 (2), I698 (13)
**Other vascular (184 deaths)**	E115 (23), E145 (24), I260 (2), I269 (46), I710 (3), I712 (1), I713 (4), I714 (1), I718 (2), I719 (1), I729 (1), I739 (2), I741 (1), I771 (10), I776 (1), I802 (6), I803 (1), I822 (1), I828 (1), I829 (2), I830 (1), I872 (1), I879 (1), I99X (1), K550 (41), K551 (1), K552 (1), K559 (3), K761 (1)
**Renal (1319 deaths)**	E102 (4), E112 (584), E122 (1), E142 (263), I120 (67), I129 (1), I130 (1), I131 (1), I132 (17), N002 (1), N039 (22), N049 (1), N059 (2), N10X (2), N12X (7), N142 (1), N151 (7), N170 (1), N179 (58), N180 (8), N185 (15), N189 (110), N19X (26), N200 (6), N201 (1), N289 (1), N390 (109), Q619 (1), Y841 (1)
**Infectious (952 deaths)**	A047 (1), A060 (1), A090 (2), A099 (28), A09X (10), A162 (8), A165 (1), A169 (2), A170 (1), A180 (1), A181 (1), A182 (1), A188 (1), A199 (3), A415 (1), A419 (140), A483 (1), A498 (1), A810 (2), A86X (2), B200 (1), B201 (1), B206 (2), B207 (1), B208 (5), B210 (1), B227 (1), B238 (4), B24X (5), B441 (1), B690 (2), B909 (1), B948 (1), G009 (3), G039 (4), G049 (6), G060 (1), H440 (1), J069 (1), J09 (3), J111 (1), J151 (3), J157 (1), J159 (13), J180 (46), J181 (22), J182 (1), J189 (291), J209 (3), J22X (14), J391 (1), J852 (1), J869 (4), K052 (1), K222 (1), K223 (2), K228 (1), K251 (3), K254 (5), K255 (5), K256 (1), K259 (3), K264 (5), K265 (2), K269 (1), K274 (3), K275 (1), K290 (5), K291 (2), K292 (1), K295 (3), K297 (1), K318 (6), K352 (4), K353 (3), K358 (1), K359 (1), K572 (2), K578 (3), K579 (8), K610 (1), K611 (1), K650 (4), K658 (1), K659 (40), K920 (4), K921 (1), K922 (84), L021 (3), L022 (5), L023 (1), L024 (1), L031 (2), L038 (1), L039 (3), L089 (10), L899 (3), L89X (4), L905 (1), L984 (6), M009 (1), M600 (2), M725 (1), M726 (10), M798 (22), M869 (1), N410 (1), N498 (6), N499 (1), N719 (1), N739 (1), N764 (1)

Webtable 2: Death rate ratios associated with previously diagnosed and undiagnosed diabetes for mortality from vascular, renal, and infectious causes (separately and combined) at ages 35-74 years, by A) duration of diabetes and B) HbA1c - Comparison of 'conventional' 95% confidence intervals with 'group-specific' 95% confidence intervals*

	Vascular		Renal		Infectious		Any vascular, renal or infectious cause	
	RR ('conventional' CI)	RR ('group-specific' CI)	RR ('conventional' CI)	RR ('group-specific' CI)	RR ('conventional' CI)	RR ('group-specific' CI)	RR ('conventional' CI)	RR ('group-specific' CI)
No diabetes	1.0 (-)	1.0 (0.9-1.1)	1.0 (-)	1.0 (0.9-1.1)	1.0 (-)	1.0 (0.9-1.1)	1.0 (-)	1.0 (1.0-1.1)
Undiagnosed diabetes	2.2 (1.8-2.5)	2.2 (1.9-2.5)	8.0 (6.4-10.1)	8.0 (6.7-9.6)	2.8 (2.2-3.5)	2.8 (2.2-3.4)	3.0 (2.7-3.4)	3.0 (2.8-3.4)
**A. Previously diagnosed diabetes, by duration (and standardised for HbA1c)**								
<5 years	3.1 (2.6-3.6)	3.1 (2.7-3.6)	13.6 (11.0-16.9)	13.6 (11.5-16.1)	3.7 (2.9-4.7)	3.7 (3.0-4.6)	4.5 (4.0-5.0)	4.5 (4.1-5.0)
≥5 to <10 years	3.8 (3.4-4.3)	3.8 (3.5-4.3)	24.2 (20.5-28.6)	24.2 (22.0-26.6)	5.2 (4.4-6.2)	5.2 (4.5-6.0)	6.6 (6.1-7.1)	6.6 (6.2-7.0)
≥10 years	6.3 (5.5-7.2)	6.3 (5.6-7.1)	48.5 (40.7-57.7)	48.5 (43.5-54.1)	8.0 (6.5-9.7)	8.0 (6.7-9.5)	11.7 (10.7-12.7)	11.7 (10.8-12.5)
**B. Previously diagnosed diabetes, by HbA1c (and standardised for duration)**								
<9%	3.2 (2.8-3.6)	3.2 (2.9-3.6)	17.3 (14.5-20.7)	17.3 (15.4-19.5)	4.2 (3.5-5.1)	4.2 (3.6-4.9)	5.2 (4.8-5.7)	5.2 (4.8-5.6)
≥9 to <11%	4.4 (3.8-5.0)	4.4 (3.9-5.0)	22.6 (18.8-27.2)	22.6 (19.9-25.7)	5.0 (4.0-6.2)	5.0 (4.1-6.0)	6.8 (6.2-7.4)	6.8 (6.2-7.4)
≥11%	5.6 (4.9-6.4)	5.6 (5.0-6.3)	41.2 (34.9-48.6)	41.2 (37.4-45.3)	7.6 (6.3-9.2)	7.6 (6.5-8.9)	10.5 (9.7-11.5)	10.5 (9.9-11.3)

* The 'conventional' confidence intervals (CI) reflect the uncertainty in the death rate ratio (RR) for the given group compared to the (arbitrary) reference group of participants without diabetes at recruitment (who are assigned the RR of 1.0). By contrast, 'group-specific' confidence intervals are shown for every death RR (including the reference group) and reflect the variance of the log risk only in that one group. These group-specific confidence intervals have the advantage that, if desired, they can be used to calculate a 95% confidence interval for <u>any pairwise comparison</u> (e.g., those with previously diagnosed diabetes <5 years versus those with undiagnosed diabetes).

Webtable 3: Baseline characteristics among men and women aged 75-84 without chronic disease at recruitment (other than diabetes)

	No diabetes (n=5413)	Undiagnosed diabetes (n=498)	Previously diagnosed diabetes (n=1599)	Previously diagnosed diabetes, by duration			Previously diagnosed diabetes, by HbA_{1c}, %			Overall (n=7510)
				<5 years (n=272)	≥5 to <10 years (n=572)	≥10 years (n=755)	<9% (n=1107)	≥9 to <11% (n=318)	≥11% (n=174)	
**Age and sex**										
Age, years	79 (3)	79 (3)	79 (3)	79 (3)	79 (3)	79 (3)	79 (3)	79 (3)	79 (3)	79 (3)
Male	2092 (39%)	181 (36%)	531 (33%)	80 (29%)	198 (35%)	253 (34%)	360 (33%)	111 (35%)	60 (34%)	2804 (37%)
**Diabetes duration and severity**										
Duration of diabetes, years*	-	-	14 (10)	2 (2)	7 (3)	22 (7)	13 (10)	14 (10)	15 (10)	-
Onset age < 35 years and insulin use at baseline †	-	-	3 (<0.5%)	0 (0%)	0 (0%)	3 (<0.5%)	3 (<0.5%)	0 (0%)	0 (0%)	-
Mean HbA_{1c}, %	5.6 (0.4)	7.9 (1.8)	8.1 (2.1)	7.8 (2.2)	8.0 (2.1)	8.3 (2.1)	7.0 (1.0)	9.9 (0.6)	12.4 (1.3)	6.3 (1.6)
Median (IQR) HbA_{1c}, %	5.6 (5.4-5.8)	7.2 (6.7-8.4)	7.6 (6.5-9.5)	7.1 (6.3-9.0)	7.5 (6.5-9.3)	8.0 (6.7-9.7)	6.9 (6.2-7.7)	9.8 (9.4-10.3)	12.0 (11.4-13.0)	5.7 (5.4-6.4)
HbA_{1c} ≥ 9.0% **	-	92 (18%)	492 (31%)	72 (26%)	163 (28%)	257 (34%)	0 (0%)	318 (100%)	174 (100%)	584 (8%)
**Socio economic status and smoking**										
Resident of Coyoacán	1890 (35%)	138 (28%)	498 (31%)	105 (39%)	140 (24%)	253 (34%)	353 (32%)	85 (27%)	60 (34%)	2526 (34%)
Resident of Iztapalapa	3523 (65%)	360 (72%)	1101 (69%)	167 (61%)	432 (76%)	502 (66%)	754 (68%)	233 (73%)	114 (66%)	4984 (66%)
University or college educated	193 (4%)	21 (4%)	46 (3%)	6 (2%)	18 (3%)	22 (3%)	36 (3%)	6 (2%)	4 (2%)	260 (3%)
Current smoker	787 (15%)	70 (14%)	183 (11%)	29 (11%)	63 (11%)	91 (12%)	126 (11%)	41 (13%)	16 (9%)	1040 (14%)
**Anthropometry and blood pressure**										
BMI, kg/m²	27.4 (4.6)	28.9 (4.4)	27.8 (4.6)	28.4 (4.7)	28.1 (4.5)	27.2 (4.6)	27.9 (4.6)	27.8 (4.5)	26.8 (4.4)	27.6 (4.6)
Systolic/diastolic blood pressure, mmHg	138/85 (19/11)	138/85 (18/11)	139/85 (19/11)	139/85 (19/11)	139/85 (19/11)	140/85 (19/11)	139/85 (19/11)	140/85 (19/11)	138/85 (20/12)	138/85 (19/11)
**Antidiabetes medication**										
Any antidiabetes	-	-	1309 (82%)	196 (72%)	462 (81%)	651 (86%)	881 (80%)	274 (86%)	154 (89%)	-
Insulin	-	-	140 (9%)	5 (2%)	21 (4%)	114 (15%)	68 (6%)	53 (17%)	19 (11%)	-
Biguanide (eg, metformin)	-	-	239 (15%)	39 (14%)	72 (13%)	128 (17%)	159 (14%)	44 (14%)	36 (21%)	-
Sulphonylurea	-	-	1101 (69%)	174 (64%)	423 (74%)	504 (67%)	755 (68%)	217 (68%)	129 (74%)	-
Other antidiabetes	-	-	24 (2%)	2 (1%)	10 (2%)	12 (2%)	19 (2%)	2 (1%)	3 (2%)	-
**Other long-term medication**										
Any antihypertensive	1747 (32%)	151 (30%)	681 (43%)	108 (40%)	250 (44%)	323 (43%)	492 (44%)	132 (42%)	57 (33%)	2579 (34%)
Renin-angiotensin system inhibitor	1252 (23%)	109 (22%)	528 (33%)	77 (28%)	193 (34%)	258 (34%)	381 (34%)	104 (33%)	43 (25%)	1889 (25%)
Other antihypertensive	645 (12%)	53 (11%)	186 (12%)	34 (13%)	67 (12%)	85 (11%)	139 (13%)	31 (10%)	16 (9%)	884 (12%)
Any antithrombotic	285 (5%)	31 (6%)	68 (4%)	13 (5%)	24 (4%)	31 (4%)	49 (4%)	13 (4%)	6 (3%)	384 (5%)
Any lipid lowering	27 (<0.5%)	2 (<0.5%)	17 (1%)	4 (1%)	8 (1%)	5 (1%)	12 (1%)	4 (1%)	1 (1%)	46 (1%)

Data are mean (SD) or n (%), unless otherwise stated.
* Estimated from age at recruitment and decade of diagnosis. † Suggestive of type 1 diabetes. ** HbA1c values above 9 reflect poor glycaemic control.
